# Parasympathetic preganglionic cardiac motoneurons labeled after voluntary diving

**DOI:** 10.3389/fphys.2014.00008

**Published:** 2014-01-28

**Authors:** W. Michael Panneton, A. Michael Anch, Whitney M. Panneton, Qi Gan

**Affiliations:** ^1^Department of Pharmacological and Physiological Science, St. Louis UniversitySt. Louis, MO, USA; ^2^Department of Psychology, St. Louis UniversitySt. Louis, MO, USA

**Keywords:** diving response, autonomic reflex, bradycardia, rostral ventrolateral medulla, cFos, visceral sensation

## Abstract

A dramatic bradycardia is induced by underwater submersion in vertebrates. The location of parasympathetic preganglionic cardiac motor neurons driving this aspect of the diving response was investigated using cFos immunohistochemistry combined with retrograde transport of cholera toxin subunit B (CTB) to double-label neurons. After pericardial injections of CTB, trained rats voluntarily dove underwater, and their heart rates (HR) dropped immediately to 95 ± 2 bpm, an 80% reduction. After immunohistochemical processing, the vast majority of CTB labeled neurons were located in the reticular formation from the rostral cervical spinal cord to the facial motor nucleus, confirming previous studies. Labeled neurons caudal to the rostral ventrolateral medulla were usually spindle-shaped aligned along an oblique line running from the dorsal vagal nucleus to the ventrolateral reticular formation, while those more rostrally were multipolar with extended dendrites. Nine percent of retrogradely-labeled neurons were positive for both cFos and CTB after diving and 74% of these were found rostral to the obex. CTB also was transported transganglionically in primary afferent fibers, resulting in large granular deposits in dorsolateral, ventrolateral, and commissural subnuclei of the nucleus tractus solitarii (NTS) and finer deposits in lamina I and IV-V of the trigeminocervical complex. The overlap of parasympathetic preganglionic cardiac motor neurons activated by diving with those activated by baro- and chemoreceptors in the rostral ventrolateral medulla is discussed. Thus, the profound bradycardia seen with underwater submersion reinforces the notion that the mammalian diving response is the most powerful autonomic reflex known.

## Introduction

Motor control of the heart is via a two neuron efferent system linked in a peripheral ganglion. Thus, there are preganglionic and postganglionic neurons innervating the heart similar to the autonomic nervous system's innervation of other viscera. It is well known that the parasympathetic component of heart control has preganglionic cardiac neurons located in the medulla while postganglionic neurons are found close to the heart itself. The consensus for the location of preganglionic cardiac motoneurons has shifted over the years (see Hopkins et al., 1996; Panneton et al., 1996; Hopkins and Armour, 1998; Hsieh et al., 1998 for reviews), with most investigators now concluding that most cardiac motoneurons are found in the external formation of the nucleus ambiguus in the ventrolateral medulla. Axons from these neurons travel peripherally with the vagus nerve; a bradycardia is induced when such fibers are activated. A functional heterogeneity has been speculated (see Blinder et al., 2007) for preganglionic parasympathetic cardiac motor neurons; we wished to determine if medullary cardiac motor neurons physiologically-activated by diving underwater are segregated in the medulla.

However, labeling of preganglionic cardiac motoneurons is problematic when using retrograde transport techniques. Application of various retrograde neuronal tracers has been made directly onto nerves innervating the heart, into intrinsic cardiac ganglia, onto the heart itself near the SA node, or into the pericardial sac surrounding the heart (see Panneton et al., 1996; Corbett et al., 2003, for discussions), but the potential for contamination of nearby structures has never been eliminated. We used both Fluorogold (FG) and cholera toxin as tracers in the present study, injecting either into the pericardial fluid surrounding the heart where it is incorporated into the axon terminals of preganglionic parasympathetic neurons and transported retrogradely into brainstem neurons. We report herein on data from cases using cholera toxin, since the results were more consistent and Corbett et al. (2003) suggested that spurious labeling is limited using this tracer after pericardial injections. Our data is reinforced by also labeling such neurons with cFos after underwater diving, potentially eliminating spurious labeling of preganglionic esophageal and tracheal neurons that are the usual candidates of contamination.

Activation of parasympathetic preganglionic cardiac motoneurons is dramatic during underwater diving in the rat, inducing a marked bradycardia (Panneton et al., 2010a,b, 2012; McCulloch et al., 2010; Panneton, 2013) that is maintained during submersion. Similar cardiorespiratory responses, including an abrupt and dramatic bradycardia, also is induced in rats to nasal stimulation with irritant vapors (McRitchie and White, 1974; White et al., 1974, 1975; Panneton, 1991b; Gieroba et al., 1994; Panneton and Yavari, 1995; Yavari et al., 1996; McCulloch and Panneton, 1997; McCulloch et al., 1999a,b; Panneton et al., 2010b). We recently reported on neurons activated during underwater submersion in voluntarily diving rats with the aid of the cFos technique (Panneton et al., 2012), but such studies allow no distinction as to the function of these activated neurons. In the present study we determine which parasympathetic preganglionic cardiac motoneurons marked by a retrograde tracer also showed diving-induced cFos activation. Such procedures allow for a qualitative assessment of neurons activated by a behavior. We show that most cardiac motoneurons physiologically-activated by diving and showing cFos were found in the rostral ventrolateral medulla.

## Materials and methods

Seven adult (~275–401 g) and 13 weanling (70–90 g) Sprague-Dawley male rats were obtained commercially (Harlan, Indianapolis, IN) and used in this study. Weanling rats were used to introduce them early in life to both water and the training protocols and as a means to limit cost. All protocols were approved by the Animal Care Committee of Saint Louis University and followed the guidelines of the National Institutes of Health Guide for Care and Handling of Laboratory Animals.

### Diving physiology

The young rats learned to swim and dive in water just after weaning through a maze constructed of Plexiglas™ (McCulloch and Panneton, 2003). The rats first negotiated the maze by swimming on the surface of the water to the finish area; the length of their swim was increased gradually (~50 cm/day) through the 5 m path. Once they were familiar with the route, they were placed in the start gate (with its exit below water level) and then trained to dive underwater for increasingly longer distances. Distances were increased by placing horizontal plastic cover plates (from 40 to 100 cm long) over the water chute, preventing early surfacing. Increments were 40–50 cm/training day over 6–10 trials/day/rat. Morning trials repeated distances of the previous training period while the distance of afternoon trials were lengthened. Thus, early trials in the training period had the rats dive initially followed by swimming to the finish area while trials later in the training period had the rats dive through the whole maze to the finish area. When placed in the start area, the rats voluntarily initiated their own dives to reach the finish area. We found that sometimes rats would get confused while making the turns underwater in the maze and revert back to the start gate. Most such confusion surprisingly was eliminated by drawing arrows on the tank pointing toward the proper direction (similar to one-way street signs). The rats appeared unstressed (McCulloch et al., 2010) either by the training protocol or exposure to water, and their willingness to dive voluntarily was clear when they swim underwater less than the 5 m length. [It is of interest that arterial partial pressures of oxygen, carbon dioxide, and oxygen saturation change dramatically only after the first ~15 s of underwater submersion (Panneton et al., 2010a), a time point consistent with all the trials in this study.] No external reward was used during the training protocol, but the rats may have been rewarded by an experience of “freedom” during training when they were allowed to run freely between cages placed on a table.

The immature rats were trained 5 days/week for 5–6 weeks to learn to both swim and dive underwater through a maze (McCulloch and Panneton, 2003). We suspect however that training could be started on slightly older animals and thus decrease the waiting time until they reached an appropriate weight to implant the transmitters. Once these rats reached 230–290 g, they were anesthetized with ketamine/xylazine (60/40 mg/kg; IP) and the catheter of a biotelemetric transmitter (Model PA-C40; Data Sciences International, DSI; St. Paul, MN USA) inserted into their femoral arteries (per a video supplied by the manufacturer); the transmitter itself was implanted in their abdominal cavities for the monitoring of pulsatile blood pressure in awake, spontaneously active animals. The rats were given buprenorphine (IP; 0.1 ml/100 g) post-operatively and healed for at least 5 days without training. After 5–18 days, retrograde tracers were injected into the rats' pericardial cavity, the rats rested for 3–4 more days, and then their diving behavior monitored for experimental data. Fortunately they did not forget their willingness either to swim or submerge underwater. Cardiovascular data were obtained from all rats both prior to, during, and after experimental submersion trials. The transmitter's broadcast was intercepted with a radio receiver—a hand held “wand” which followed the diving rat (Model RLA3000; DSI), relayed to a Calibrated Pressure Analog Adaptor (Model R11CPA; DSI), and transferred through an A-D interface (1401 plus; Cambridge Electronic Design, CED; Cambridge UK), stored in the computer, and analyzed using Spike 2 software (CED). Systolic, diastolic and mean arterial blood pressure (MABP) was calculated and heart rate (HR) was determined by counting peaks of systolic pressure. Control data was taken from injected rats in the start gate prior to underwater submergence while experimental data was obtained while the rats were diving. Also, we assumed that the rats did not attempt to breathe while underwater since they showed no difficulty in breathing after their experience—all readily dove underwater multiple times and none drowned during submergence. On the day of the experiment, the rats dove voluntarily three times within 11.5–13.5 min (avg. 12.7 min) and their hemodynamics monitored.

### Data analysis

Means and standard errors (M ± S.E.) were determined for experimental and control data from the cardiovascular parameters. The three dives were averaged per animal and a grand mean calculated. HR and MABP during diving were compared to data taken just prior to submersion in all experimental rats and compared for significance (SPSS software; v. 13) using the Independent Samples *T*-test. Significance was calculated as *p* < 0.05.

### Immunohistochemistry

Initially, four adult rats were anesthetized with ketamine/xylazine (60/40 mg/kg; IP) and their thorax shaved prior to surgery. An endotracheal tube was inserted orally and the rats ventilated mechanically. The rats were secured supine, and a right thoracotomy performed between the 2nd and 4th rib and the opening expanded with retractors. After locating the pericardium over the right atrium, between 10–20 μl of 5% FG (Fluorochrome, Inc., Denver, CO, USA) in 0.2 M cacodylate buffer mixed with vital dye Fast Green was injected into the pericardium via a 10 μl Hamilton syringe to which a fine glass tip (20–35 μm) had been attached. The green vital dye allowed for qualitative assessment of the injection and was deemed successful if the dye was contained within the pericardium. Sutures were placed through the intercostal muscles, the lungs hyperinflated, and then knotted securely over the incision. Their skin was closed and the rats received subcutaneous injections of buprenorphine (0.1 ml/100 g) postoperatively.

After 3–4 days the rats were deeply anesthetized (Sleepaway, 40 mg/kg; IP) and perfused through the heart with a peristaltic pump first with a saline-procaine solution, followed immediately by a fixative of 4% paraformaldehyde and 3% sucrose in 0.1 M sodium phosphate buffer (PB; pH 7.3). Brains were removed and refrigerated in the fixative with 20% sucrose at 4°C. The brains were blocked in the transverse plane using a precision brain slicer prior to cutting frozen transverse sections (40 μm) with a microtome.

Every third section was washed three times with 0.1 M PB for 10 min, and then in 0.1 M PB with 0.3% Triton for at least 5 min. Sections then were processed immunohistochemically overnight with antibodies against FG in buffer with 0.3% Triton containing rabbit anti-FG (1:20,000; Chemicon, Temecula, CA, USA) on a shaker at room temperature. The following morning, the sections were washed in PB with 0.3% Triton and incubated for 1 h in a solution containing goat anti-rabbit immunoglobulin (Sigma-Aldrich Corp., St. Louis, MO, USA) at a dilution of 1:400. The sections then were incubated in Vectastain ABC Elite solution (1:200; Vector Laboratories, Burlingame, CA, USA) for 1 h, washed in three rinses of PB, and reacted with diaminobenzidine dihydrochloride (DAB) intensified with nickel ammonium sulfate for 4–10 min. Hydrogen peroxide (0.06%) catalyzed the reaction.

However, the retrograde staining of preganglionic cardiac motoneurons was minimal using FG, so pericardial injections of Cholera Toxin B Subunit (CTB) Conjugate (List Laboratories, Inc.) were made in the other 16 rats (13 juvenile and 3 adult), but only after the juvenile rats first had been trained to dive underwater (vide supra). Upon reaching at least 230 g, these juvenile rats were instrumented with telemetric transmitters (vide supra) and after 5–18 days subjected to thoracotomy as described previously. Between 5–20 μl of 1% CTB was injected into the pericardial sac as above, with most injections between 8–11 μl. After a 3–4 days transport period, the injected rats dove underwater 3 times over a period of approximately 13 min and were perfused as above after 2 h. In 5/13 of these cases the thin pericardium developed a tear, and green dye spread into the pleural cavity. Thus, in three other cases in adult rats the CTB-Fast Green injection was placed directly into the pleural cavity as control. The brains of all rats were removed and subsequently sectioned frozen at 40 μm on a sliding microtome. One series of sections was first processed immunohistochemically with antibodies against CTB (vide infra) to determine reliable transport. Another series was then processed for cFos and CTB immunohistochemistry.

After rinsing (vide supra), every third section was processed immunohistochemically overnight with antibodies against Fos (rabbit polyclonal IgG for *c-fos* p62; 1:20,000; Santa Cruz Biotechnology, Inc.) mixed in 0.1 M PB with 0.3% Triton. On the following day, the sections were washed, incubated for 1 h in goat anti-rabbit biotinylated secondary IgG (1:500; Vector Labs), washed again, and then incubated in an ABC complex (Vectastain Elite; Vector Laboratories) for another hour. The Fos antigen was visualized in the brainstem with the chromogen diaminobenzidine (DAB) enhanced with nickel ammonium sulfate, resulting in a black precipitate in nuclei of immunolabeled cells. After rinsing, the sections were then incubated in Avidin-D (12.5 mg/ml; Vector Laboratories) for 30 min, rinsed in 0.1 M phosphate buffer with 0.3% Triton, incubated in d-Biotin (25 mg/ml) for another 30 min, and then rinsed again in buffer. The sections were soaked overnight in goat anti-CTB subunit (List Laboratories, Inc.), and then processed with donkey anti-goat secondary antibody. Visualization of the 2nd antigen was done histochemically similar to that above with the exception that nickel ammonium sulfate was omitted, resulting in a brown reaction product. The sections from all groups were then rinsed, mounted serially on gelatinized slides and air-dried. Slides then were counterstained with Neutral Red, dehydrated in alcohols, defatted in xylenes, and coverslipped with Permount.

Neurons contained black nuclei when stained with a single antigen against cFos and had brown cytoplasm when stained only for CTB; double-labeled neurons appeared as cells with black-labeled nuclei and brown cytoplasm. Neurons were visualized with brightfield optics (Nikon E800) and photographed with a digital camera (MicroImager II) and Northern Eclipse Software (Empix, Inc.). Sections were drawn with a Nikon E600 microscope and Neurolucida software (MicroBrightField, Inc.). The photomicrographs were standardized using levels, brightness and contrast in Adobe Photoshop software (v.7) and aligned in Adobe Illustrator software (v.11) for figures. Figure 3A is a photomontage of 23 individual figures stitched together by Microsoft Image Composite Editor (open source; http://research.microsoft.com/en-us/um/redmond/groups/ivm/ice/), an advanced panoramic image stitcher. Nomenclature and abbreviations are from a stereotaxic rat atlas (Paxinos and Watson, 1998).

## Results

**Figure 1:**
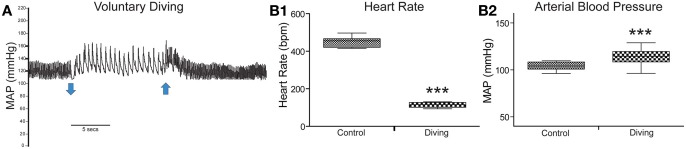
**Cardiovascular responses to underwater submersion of voluntarily diving rats.** The abrupt bradycardia and increase in mean arterial pressure (MAP) is seen in **(A)** upon submergence (down arrow), which immediately reverts to normal rhythms after emergence from the water (up arrow). Compilation of trials (*n* = 24) from eight rats showing the 80% drop in heart rate (**B1**; HR, *p* < 0.001) and increase in MAP (**B2**, *p* < 0.001) after underwater submersion. Rats exhibit an almost invariant diving response. ^***^p < 0.001.

Results for diving physiology were calculated for 8/13 rats that had pericardial injections of CTB without tearing this delicate membrane; data from the others were not considered since their pericardial membranes developed tears during injection. All rats showed an abrupt and dramatic bradycardia upon underwater submergence with an increase in arterial blood pressure (Figure 1A). Mean HR prior to voluntary diving was 465 ± 4 bpm while MAP was 114 ± 1.6 mmHg; this data served as control. Upon underwater submersion, HR immediately dropped significantly (*p* < 0.001) to 95 ± 2 bpm, an 80% reduction (Figure 1B1), while MAP rose significantly (*p* < 0.001) to 127 ± 1 mmHg (Figure 1B2).

**Figure 2:**
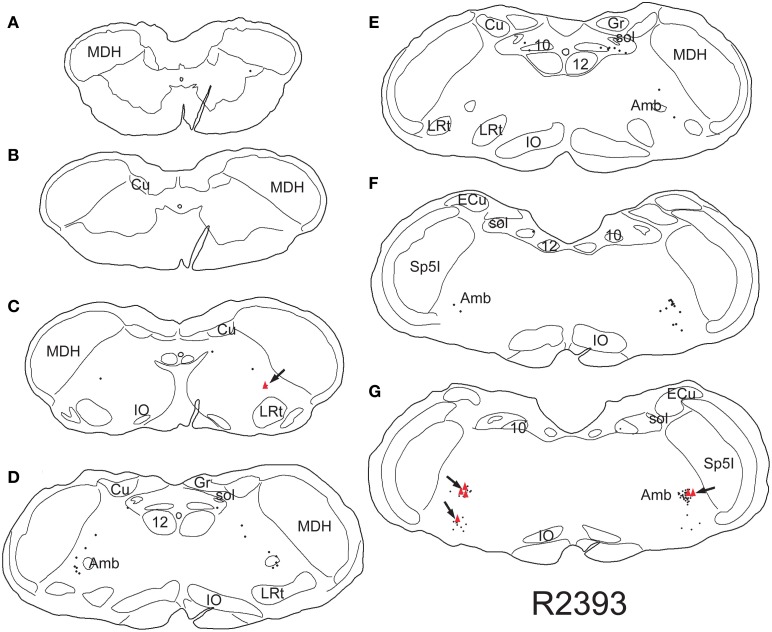
**Line drawings illustrating neurons labeled singly with CTB (small dots) or doubly with CTB and cFos (red triangles) in a rat trained to dive underwater and receiving a pericardial injection of CTB.** While single labeled neurons were found mostly in the reticular formation, especially in the external formation of the nucleus ambiguus, most double-labeled neurons are found in the rostral ventral lateral medulla (arrows). Thus those activated by diving are generally found in the RVLM. Abbreviations: Amb, nucleus ambiguus; Cu, cuneate nucleus; ECu, external cuneate nucleus; Gr, gracile nucleus; IO, inferior olivary nucleus; LRt, lateral reticular nucleus; MDH, medullary dorsal horn; Sp5I, spinal trigeminal nucleus, pars interpolaris; sol, solitary tract; sp5, spinal trigeminal tract; 10, dorsal motor nucleus of the vagus nerve; 12, hypoglossal motor nucleus.

**Figure 3:**
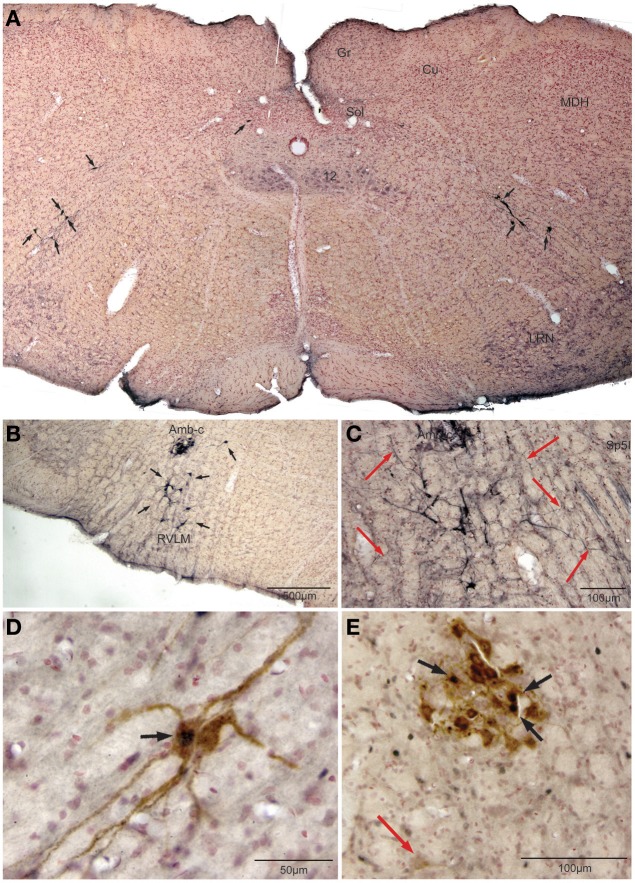
**Photomicrographs illustrating retrograde labeling of parasympathetic preganglionic cardiac motor neurons in the medulla of the rat after pericardial injections of cholera toxin B (CTB) and their activation by diving.** Photomontage of a medullary section caudal to the obex **(A)** showing the distribution of caudal preganglionic cardiac motoneurons (arrows) along an oblique line from the ventrolateral part of the dorsal motor nucleus of the vagus nerve toward the nucleus ambiguus. Such distribution is similar to that described in other mammals. Most neurons rostral to the obex **(B,C,E)** were retrogradely-labeled adjacent or ventral to the compact formation of nucleus ambiguus (Amb-c). Note the long dendrites of neurons ventral to Amb-c (**C**, red arrows) extending into the reticular formation, many surrounding Amb-c or approaching the spinal trigeminal nucleus, pars interpolaris (Sp5I). Double labeling of preganglionic cardiac motor neurons after underwater submersion with both cFos (black immunolabeled nuclei) and retrograde transport of CTB (brown cytoplasmic labeling) is shown in **(D,E)**. Two retrogradely-labeled neurons in the caudal medulla are seen in **(D)**, one of which was activated by underwater submersion (arrow); note the long obliquely orientation of the dendrites. While some Amb-c neurons were retrogradely-labeled **(E)** after pericardial injections, neurons labeled with cFos were found on its periphery, some of which were double-labeled (black arrows). A single retrogradely labeled neuron also is shown more ventrally (red arrow).

Retrogradely labeled neurons (average/case = 167 neurons) were noted bilaterally in the brainstem (Figure 2) after CTB injections into the pericardium. Most were contained in the medulla, but some were found in the C1 cervical segment of the rostral spinal cord (Figure 2A). Most of the retrogradely labeled neurons found caudal to the obex were of medium size in the ventrolateral reticular formation (Figure 3A), generally extending along an oblique line from the dorsal motor nucleus of the vagus nerve to nucleus ambiguus (Figure 3A, arrows), where many were included in its external formation. These neurons were elongated along this oblique line and contained long dendrites oriented similarly (Figures 3A,D). A few neurons were in the dorsal vagal nucleus itself; these were small with little dendritic labeling. Above the obex, those ventral to the nucleus ambiguus were the largest and intermixed in the area of the C1 catecholaminergic group. They were pyramidal or multipolar in shape (Figure 3B, arrows) with extensive dendrites (Figure 3C, arrows) extending laterally toward the trigeminal sensory complex and around the compact formation of the nucleus ambiguus. Retrogradely labeled neurons within the compact formation of nucleus ambiguus were multipolar with short dendrites that seldom were seen beyond the vicinity of the neuronal cell body (Figure 3E).

Nine percent of retrogradely-labeled neurons were positive for cFos and CTB and 74% of these were found rostral to the obex (Figure 2). Double-labeling in these neurons was prominent (Figures 3D,E; black arrows), but we suspect more nuclei were labeled with cFos but obscured by dense cytoplasmic concentrations of CTB. Nevertheless, almost three quarters of double-labeled neurons were found rostral to the obex and just caudal to the facial motor nucleus (Figure 2), both surrounding the compact nucleus ambiguus (Figure 3E) as well as in the rostral ventrolateral medulla (Figures 3B,C).

Only half as many neurons (*n* = 88/case) were retrogradely labeled after injections of cholera toxin into the pleural cavity (*n* = 3). The distribution of neurons after pleural injections generally was similar to that after injection into the pericardial cavity, but we considered the staining to be less intense. We also noted that more retrogradely labeled neurons were found in the dorsal motor nucleus of the vagus nerve after injections into the pleural cavity, especially rostrally.

**Figure 4:**
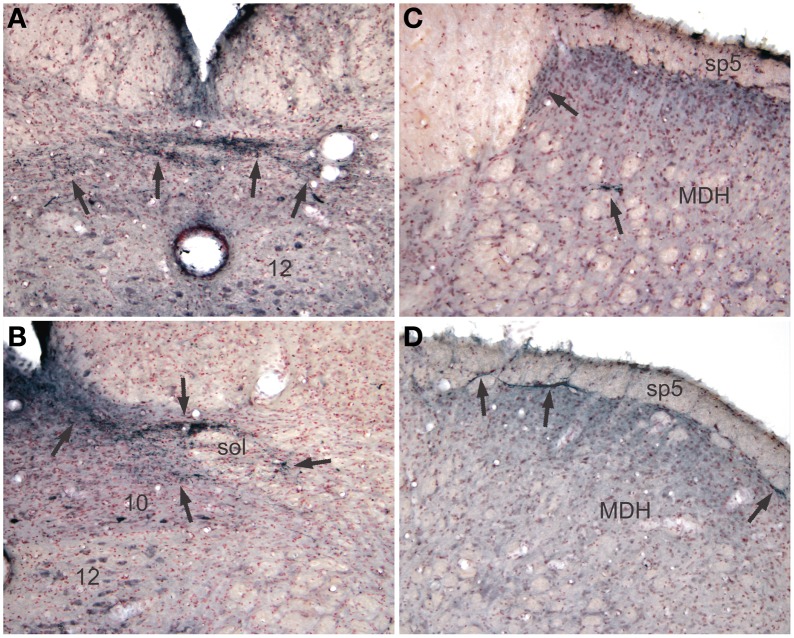
**Pericardial injections of CTB also induced transganglionic transport in sensory fibers.** Note black precipitate (arrows) in the commissural subnucleus (**A**, arrows) and dorsolateral and ventrolateral subnuclei (**B**, arrows) of the nucleus tractus solitarii; such label mimics that seen after labeling primary afferent fibers innervating baroreceptors and the trachea. Labeling of sensory afferent fibers was also noted in the C_1_ spinal segment **(C)** and medullary dorsal horn (MDH) of the trigeminal sensory complex **(D)** in laminae I and IV–V (arrows).

Reaction product, reminiscent of transganglionic transport in sensory fibers, also was seen in all cases with injections into the pericardial cavity, but not after injections into the pleural cavity. Label consisting of large granules of reaction product was noted bilaterally in the commissural subnucleus of the nucleus tractus solitarii (NTS: Figure 4A, arrows) and extended rostrally to the obex where it was found bilaterally in the dorsolateral, dorsomedial, and ventrolateral subnuclei of the NTS (Figure 4B). Transganglionic label also was seen bilaterally in the dorsomedial third of the caudal medullary dorsal horn, possibly from labeled fibers exiting the caudal tractus solitarii and traveling laterally. A finer label was found in lamina I bilaterally (Figure 4D, arrows) while a mixed label of both small and large granules were seen in laminae IV–V bilaterally (Figure 4C).

## Discussion

Pericardial injections of cholera toxin were made in rats trained to dive underwater and the resultant retrograde labeling combined with cFos immunohistochemistry located parasympathetic preganglionic cardiac motor neurons activated by voluntary diving. All rats showed a drop in HR of approximately 80%, similar to all diving trials published previously by us in this rodent (Panneton et al., 2010a,b, 2012; Panneton, 2013). Preganglionic cardiac motoneurons have been described by many as rather isolated in the medulla, having no clear nuclear bounds. However, a small number of these neurons induce marked adjustments to HR when activated/depressed after microinjections of transmitters (McAllen and Spyer, 1976; Agarwal and Calaresu, 1991; Chitravanshi et al., 1991; Chitravanshi and Calaresu, 1992; Ruggeri et al., 2000), suggesting the powerful influence of a few over cardiac rhythms. Although parasympathetic preganglionic cardiac motor neurons were double-labeled from the spinomedullary junction to the caudal facial nucleus herein, most double-labeled motoneurons were found in the rostral ventrolateral medulla. This location overlaps with that of other somatoautonomic cardiorespiratory reflex circuits, and provides a locus for the radical adjustment to normal homeostatic rhythms seen during the diving response.

### Technical considerations

Numerous investigators have injected various retrograde tracers into physiologically-defined cardiopulmonary branches of the vagus, into subpercardial postganglionic plexi, or into the pericardium itself to determine the origin of parasympathetic preganglionic cardiac motor neurons. Our intrapericardial injections of FG were basically ineffective since few preganglionic cardiac neurons were retrogradely labeled, similar to observations of others (Mendelowitz and Kunze, 1991; Irnaten et al., 2003). FG was used effectively in one study (Aicher et al., 2002), however, but the volume injected by them greatly exceeded that used in the present study. However, numerous others have used either bound or native horseradish peroxidase molecules, fluorescent dyes, or cholera toxin effectively. While the conclusions of recent investigations using these various methods are similar, a lingering problem plaguing nearly all intrathoracic injections of tracers is that of spread to nearby structures (see Corbett et al., 1999, for discussion). We previously had injected the plexi containing postganglionic neurons in the muskrat (Panneton et al., 1996), but were troubled that neurons in the compact formation of the nucleus ambiguus, which contains numerous esophageal motoneurons (Bieger and Hopkins, 1987; Neuhuber et al., 2006; Hou et al., 2009; McGovern and Mazzone, 2010), were labeled consistently. Other investigators (Miura and Okada, 1981; Stuesse, 1982; Izzo et al., 1993) also have noted labeling in the compact formation of the nucleus ambiguus after intrathoracic injections of retrograde tracers, but Corbett et al. (2003) using CTB as a tracer concluded it only labeled cardiac motoneurons. Indeed, numerous neurophysiological studies (McAllen and Spyer, 1976, 1978; Agarwal and Calaresu, 1991, 1992; Chitravanshi et al., 1991; Chitravanshi and Calaresu, 1992; Ruggeri et al., 2000) show preganglionic cardiac neurons surrounding and just ventral to the nucleus ambiguus, similar to the present data. Nevertheless, although we agree with those stating that the compact formation of nucleus ambiguus contains esophageal motoneurons, our control injections into the pleural cavity yielded a similar distribution to those with injections into the pericardial cavity, albeit fewer neurons were labeled retrogradely and more were located in the dorsal motor nucleus of the vagus nerve. Thus, it is possible that not all retrogradely labeled neurons were parasympathetic preganglionic cardiac motor neurons.

In this regard, it is well known that cardiac pacing is tightly coupled to respiration, producing a respiratory sinus arrhythmia. Indeed, McAllen and colleagues (McAllen and Spyer, 1978; Rentero et al., 2002) showed preganglionic vagal neurons projecting into either cardiac or pulmonary branches of the vagus nerve and most neurons exiting in the cardiac branches were modulated by respiration. Although these same authors suggest that esophageal or pulmonary contamination of cardiac branches is small (McAllen and Spyer, 1976), the medullary origin of preganglionic neurons projecting to the trachea (Haxhiu and Loewy, 1996) have a similar distribution in the caudal medulla with that described herein. Thus, some of the neurons labeled herein may project to the trachea. However, although we saw numerous labeled neurons in the dorsal motor nucleus of the vagus nerve after our intrapleural control injections, there were relatively few after intrapericardial injections, suggesting the cholera toxin was contained within the pericardium (see Corbett et al., 2003). Moreover, the dorsal motor nucleus of the vagus nerve has few preganglionic cardio-inhibitory neurons (McAllen and Spyer, 1976), supporting the dearth of projections seen after our intrapericardial injections.

Moreover, our observations on the morphology of retrogradely-labeled preganglionic cardiac motoneurons in the different regions (dorsal motor nucleus of the vagus nerve, intermediate reticular formation, external formation of the nucleus ambiguus, and rostral ventral lateral medulla) align well with the morphological descriptions of others (Izzo et al., 1993; Corbett et al., 1999, 2003), suggesting specific labeling by the methods employed herein. It is unknown whether the different regional morphologies either suggest different functions of these distinct neurons or is the result of physical constraints of surrounding neuropil.

### Double-labeling of preganglionic cardiac motoneurons

We utilized cFos immunohistochemistry to mark the subset of cardiac motoneurons activated by the intense bradycardia induced by underwater submersion. We feel that when the results from both techniques are combined, parasympathetic preganglionic cardiac motor neurons activated by diving are revealed. However, only 9% of retrogradely-labeled neurons also were labeled with cFos. This may reflect the powerful action of but few cardiac motoneurons or a masking of cFos labeling by dense cytoplasmic aggregations of CTB.

Cases from our library show that the compact formation of nucleus ambiguus is labeled rarely by cFos after diving (Panneton et al., 2010b, 2012; Panneton, 2013), but is seen, however, in the semi-compact formation more caudally, potentially related to motoneurons innervating the larynx (Bieger and Hopkins, 1987; Panneton, 1991a). [It is of interest that glottal closure is induced in the diving response after nasotrigeminal stimulation (Dutschmann and Paton, 2002; Kunibe et al., 2003)]. However, cardiac neurons also can be activated via the baroreceptor and respiratory chemoreceptor reflexes, and these also may be activated during underwater submersion.

Thus, our analysis was compounded further since the diving response also increases arterial blood pressure (Figure 1), activating the baroreceptor reflex which also induces a bradycardia. ChAT-positive neurons, representing preganglionic parasympathetic neurons, were double-labeled with Fos after increases in arterial blood pressure (Okada and Miura, 1997) and had a distribution similar to those described herein (Figure 2), including many in the rostral ventrolateral medulla. Although we have shown previously that the bradycardia induced by nasal stimulation persists after increases in arterial pressure are neutralized with prazosin (Yavari et al., 1996), some preganglionic parasympathetic cardiac motor neurons double-labeled during diving herein may not have been activated by diving via trigeminal stimulation (see Panneton, 2013), but rather due to the increase in blood pressure alone.

Moreover, bradycardia also is induced when respiratory chemoreceptors are activated. However, blood gases change relatively little during the brief time (<20 s) the present rats were underwater (Panneton et al., 2010a) and chronic intermittent hypoxia does not increase Fos staining (Knight et al., 2011). Nevertheless, McAllen et al. (2011) recently have shown that convergence of reflex arcs driving the bradycardia during diving, the baroreceptor reflex and the respiratory chemoreceptor reflex occurs at the level of the parasympathetic preganglionic cardiac neurons in the medulla. This demonstrates that the same preganglionic parasympathetic cardiac motoneurons are activated by diving, baroreceptors and chemoreceptors. Thus, if neurons are labeled by both cFos and CTB markers, they probably are parasympathetic preganglionic cardiac neurons.

### Sensory transganglionic label

Sensory fibers also transported the CTB tracer transganglionically, but only after the pericardial injections. The transganglionic labeling we saw in the NTS supported early observations of HRP transport (Kalia and Mesulam, 1980) and that of pericardial injections of CTB (Corbett et al., 2005), but the function of such fibers is not known. Numerous sensory afferent fibers from the vagus nerve innervate the atria and great vessels of the heart (Foreman, 1986; Corbett et al., 2005) and it is probable that most project into the NTS. The pattern of labeling mimicked that made from peripheral baroreceptor nerves (Panneton and Loewy, 1980; Chan and Sawchenko, 1994, 1998), and may be important for sensing cardiovascular phenomena. It also may be important in modulating respiratory sinus arrhythmias, since the pattern matches the afferent labeling seen after cholera toxin injections of the trachea (Haxhiu and Loewy, 1996) and pulmonary C-fiber afferent fibers modulate preganglionic cardiac motoneurons (Wang et al., 2000; Wang and Ramage, 2001). Most of the reaction product seen herein was found in large granules, possibly boutons, and was similar to that described ultrastructurally in myelinated fibers, many of which express VGLUT1 immunoreactivity (Corbett et al., 2005). More recent data suggests a role in cardiosomatic motor reflexes (Sun et al., 2011; Liu et al., 2012).

Transganglionic labeling also was noted in lamina I and V of the caudal medullary dorsal horn and first cervical segments of the somatosensory trigeminocervical complex. The label generally was in very small granules, contrasting that seen in the NTS. Such contrasts are similar to comparisons noted between primary afferent input into laminae I and III of the MDH (Panneton et al., 2010c); lamina I receives mostly small diameter fibers while lamina III receives large myelinated fibers. This labeling was found dorsally in the trigeminocervical complex, well-known to be where primary afferent fibers carried in the mandibular division of the trigeminal nerve and rostral cervical spinal nerves terminate. It is of interest that neurons in lamina I and V of the C_1_ and C_2_ levels respond to algogenic chemicals injected into the pericardium (Qin et al., 2001, 2002; Zhang et al., 2003). All such neurons also had somatic receptive fields, similar to findings from other studies (Mørch et al., 2007), and are suggested to be important in referred pain in the jaw and neck after cardiac ischemia (Qin et al., 2001).

### Synaptic drive of cardiac motoneurons

The results of many studies suggest that parasympathetic preganglionic cardiac motor neurons seldom are clustered, making them difficult to identify without retrograde markers. However, the caveats discussed previously concerning the validity of retrograde labeling via pericardial injections must be considered. Indeed Mendelowitz et al. (Mendelowitz and Kunze, 1991; Mendelowitz, 1996; Aicher et al., 2002; Gorini et al., 2009) has shown clusters of labeled motoneurons similar to that seen in the compact formation of nucleus ambiguus. Nevertheless, work *in vitro* brainstem slices after similar retrograde labeling methods as described herein show parasympathetic preganglionic cardiac motoneurons are modulated by both glutamatergic (Willis et al., 1996; Mendelowitz, 1998; Neff et al., 1998; Corbett et al., 2003) and GABAergic/glycinergic (Wang et al., 2001b, 2003) inputs from the NTS. Preganglionic cardiac neurons also are modulated by nicotinic cholinoceptors (Wang et al., 2001a), which facilitate glutamatergic input to them (Huang et al., 2004), and numerous peptides (Agarwal and Calaresu, 1991; Ruggeri et al., 2000; Irnaten et al., 2003; Blinder et al., 2005, 2007; Hou et al., 2009) and monoamines (Izzo et al., 1993; Wang and Ramage, 2001; Skinner et al., 2002; Gorini et al., 2009). Indeed, cardiac motoneurons activated by stimulation of the trigeminal root is modulated by serotonin (Gorini et al., 2009) and acetylcholine (Gorini et al., 2010) receptors. It is unfortunate however that Mendelowitz and colleagues fail to describe from where in the medulla they take their slices; thus comparison with our results cannot be made.

### Potential pathways for a trigemino-cardiac reflex circuit

The mammalian diving response is an amalgam of three independent reflexes regulating HR, blood pressure and respiration. Innervation of paranasal areas provides an afferent link for inducing the diving response (reviewed by Panneton, 2013) and this area is innervated in part by the anterior ethmoidal nerve. We show presently that most preganglionic parasympathetic cardiac motoneurons are found in the RVLM within or near the external formation of the nucleus ambiguus, an area known to elicit bradycardia when stimulated (Ciriello and Calaresu, 1982; Stuesse and Fish, 1984). The problem remains however as to how the afferent drive is linked to the efferent output.

Since a diving bradycardia is elicited by electrically stimulating the anterior ethmoidal nerve (Dutschmann and Herbert, 1996, 1997; McCulloch et al., 1999a), it is possible that preganglionic cardiac motor neurons are modulated by direct projections into the rostral ventrolateral medulla from primary afferent fibers of this nerve (Panneton, 1991a; Panneton et al., 2006). Moreover, indirect projections from the trigeminal medullary dorsal horn are probable. Neurons in the rostral ventrolateral medulla are labeled transneuronally after HSV-1 virus injections into the anterior ethmoidal nerve (Panneton et al., 2000), and axonal swellings are seen here after injections of BDA into areas of the MDH where the anterior ethmoidal nerve terminates (Panneton, 1991a; Panneton et al., 2000, 2006). Also, the bradycardia induced by nasal stimulation is reduced after injections of lidocaine or kynurenate into similar parts of the medullary dorsal horn (Panneton and Yavari, 1995; Panneton, 2013), suggesting this somatosensory nucleus modulates cardiac activity. These studies provide a neuroanatomical substrate for both direct and indirect routes of trigeminal modulation of cardiac reflex behavior. We suggest that this reflex has but few synapses, since diving bradycardia is evoked 100% of the time in 100% of the rats tested with little variation in our hands.

Our data also promotes the rostral ventrolateral medulla as the nexus for somatoautonomic reflexes, supporting data of others (Stornetta et al., 1989; Ruggeri et al., 1995; Kawabe et al., 2007; Burke et al., 2011). Reflex circuits currently are seldom studied, yet such circuits regulate much of our behavior. Our past efforts have been directed toward defining the neural circuitry driving the dramatic bradycardia, the peripheral vasoconstriction, and apnea induced by a somatic stimulus that defies normal homeostatic parameters. Once the neural circuit for the remarkable mammalian diving response is finalized, it may provide a substrate for understanding its use in both diagnostic and therapeutic measures in humans (Leiter and Böhm, 2007; Pedroso et al., 2012; Smith et al., 2012).

### Conflict of interest statement

The authors declare that the research was conducted in the absence of any commercial or financial relationships that could be construed as a potential conflict of interest.
